# Infection of Human Monocytes with *Leishmania infantum* Strains Induces a Downmodulated Response when Compared with Infection with *Leishmania braziliensis*

**DOI:** 10.3389/fimmu.2017.01896

**Published:** 2018-01-08

**Authors:** Agostinho Gonçalves Viana, Luísa Mourão Dias Magalhães, Rodolfo Cordeiro Giunchetti, Walderez O. Dutra, Kenneth J. Gollob

**Affiliations:** ^1^Laboratório de Biologia das Interações Celulares, Departamento de Morfologia, Instituto de Ciências Biológicas, Universidade Federal de Minas Gerais, Belo Horizonte, Brazil; ^2^Instituto Nacional de Ciência e Tecnologia de Doenças Tropicais (INCT-DT), Belo Horizonte, Brazil; ^3^Núcleo de Ensino e Pesquisa, Instituto Mario Penna, Belo Horizonte, Brazil; ^4^International Center for Research, AC Camargo Cancer Center, São Paulo, Brazil

**Keywords:** *Leishmania*, monocytes, cytokines, costimulatory molecules, protozoan parasites, pathogen immunity

## Abstract

Human infection with different species of *Leishmania* leads to distinct clinical manifestations, ranging from relatively mild cutaneous (*Leishmania* braziliensis) to severe visceral (*Leishmania infantum*) forms of leishmaniasis. Here, we asked whether *in vitro* infection of human monocytes by *Leishmania* strains responsible for distinct clinical manifestations leads to early changes in immunological characteristics and ability of the host cells to control *Leishmania*. We evaluated the expression of toll-like receptors and MHC class II molecules, cytokines, and *Leishmania* control by human monocytes following short-term infection with *L. braziliensis* (M2904), a reference strain of *L. infantum* (BH46), and a wild strain of *L. infantum* (wild). The induction of TLR2, TLR9, and HLA-DR were all lower in *L. infantum* when compared with *L. braziliensis*-infected cells. Moreover, *L. infantum*-infected monocytes (both strains) produced lower TNF-alpha and a lower TNF-alpha/IL-10 ratio, resulting in a weaker inflammatory profile and a 100-fold less effective control of *Leishmania* than cells infected with *L. braziliensis*. Our results show that *L. infantum* strains fail to induce a strong inflammatory response, less activation, and less control of *Leishmania* from human monocytes, when compared with that induced by *L. braziliensis* infection. This functional profile may help explain the distinct clinical course observed in patients infected with the different *Leishmania* species.

## Introduction

A dramatic variation of clinical manifestations upon *Leishmania* infection has been known for many years, suggesting that infection with either more than one species, or variants of the same species of *Leishmania* parasites leads to distinct clinical outcomes (1–3). Genetic ancestry studies have shown that the different species of *Leishmania* currently known have evolved from a common ancestor (4). Several factors can be associated with speciation within the *Leishmania* genus, but perhaps one of the most important is the evolutionary pressure posed by contact with different hosts. The different species of *Leishmania* not only display distinct morphological and biological characteristics, but are also associated with different patterns of human disease development. Most species of *Leishmania* can cause clinical disease, ranging from a spectrum of tegumentary forms (for example, *Leishmania braziliensis*), to severe and potentially deadly visceral disease (for example, *Leishmania infantum*) (1, 3, 4). Interestingly, while distinct clinical outcomes have been associated with infection by different species of *Leishmania*, studies have also demonstrated that the same parasite species can cause distinct disease manifestations depending on the host (5) and can lead to distinct clinical manifestations in humans due to different strains from the same species (6), as well as differential stimulatory activity *in vitro* (7). Furthermore, different isolates of *L. braziliensis* have been shown to induce distinct pathology in animal models (8, 9). These findings have important implications in understanding parasite biology and also implicate the host immune response in disease evolution.

According to the WHO, there are an estimated 1.3 million total new cases of leishmaniasis observed each year in the world. Ninety percent are concentrated in only six countries, with Brazil being one of these highly endemic countries, where the tegumentary forms (cutaneous, mucosal, and disseminated) are caused mainly due to infection with *L. braziliensis* and *L. amazonensis*, and visceral disease is caused by infection with *L. infantum* (1). The tegumentary cutaneous form of leishmaniasis is characterized by the development of one or more ulcers at/near the site of infection and is associated with a robust inflammatory response that has been associated with pathology (10–17). However, it is also associated with parasite control and in association with therapy, leads to cure (3, 5). Mucosal leishmaniasis leads to destructive lesions in the naso and oropharingeal mucosa, and is associated with a vigorous production of inflammatory cytokines, such as TNF-alpha and IFN-gamma, with less regulation of the response (15, 18, 19). Different from the tegumentary forms, visceral leishmaniasis is associated with a downmodulated immune response, in which IL-10 seems to play a critical role (20–23). This is the most severe form of leishmaniasis and, if not properly identified and treated, can lead to death. This demonstrates that the immune response plays an important role in disease development, persistence and cure.

Early contact between the parasite and the host, regardless of the infecting species, involves the entry of *Leishmania* into monocytes/macrophages. This interaction relies on a number of surface receptors such as Toll-like receptors and the complement receptor CD11b (24–26), and triggers several signaling pathways that influence the immune response mounted by the monocyte/macrophage. Between *Leishmania* species, different ligands for these and other receptors are expressed and, thus, their interaction with host cells may lead to distinct activation, which could explain differences in the resulting immune response (5, 27–29). In this work, we tested the hypothesis of whether *in vitro* infection of human monocytes by different species of *Leishmania* will lead to distinct phenotypic, functional immunological, and *Leishmania* control profiles in monocytes, and whether these profiles can help explain differential clinical form development observed in human infection. Our data show that infection with *L. infantum* isolates leads to a preferential establishment of a modulatory environment and less *Leishmania* control, when compared with *L. braziliensis*, which can be associated with the immune response observed in patients with visceral and tegumentary disease, respectively, suggesting that the early events triggered by the infection with different parasite species may drive the resulting response observed in human infection.

## Materials and Methods

### Human Blood Samples and Preparation of Peripheral Blood Cells

Peripheral blood was obtained by venipuncture from a total of 12 healthy volunteers from Belo Horizonte, MG Brazil composed of, six males and six females, into tubes containing sodium heparin. The mean age of the donors was 29.4 ± 7 years of age, ranging between 21 and 45 years. This study was approved by the National Ethical Committee (CONEP # CAAE 01229212.0.0000.0049).

Peripheral blood mononuclear cells (PBMCs) were obtained as previously performed by us (30). Briefly, peripheral blood was diluted 1:1 with phosphate-buffered saline (PBS) and slowly layered over Ficoll-Paque (GE Healthcare, Piscataway, NJ, USA). Tubes were centrifuged at 200 *g* for 40 min at 20°C. After centrifugation, PBMC were harvested, washed three times by centrifugation with PBS, and resuspended in RPMI medium supplemented with antibiotics (penicillin 200 U/ml and streptomycin 0.1 mg/ml), 1 mM l-glutamine, and 10% inactivated human serum [complete RPMI media (cRPMI)]. Cell viability was assessed by trypan blue dye exclusion. Cells were counted in hemacytometer and concentration adjusted to 10^7^ cells/ml for plating in cultures.

### Parasites

Two of the parasite strains used in this work were obtained from World Health Organization repository and belonged to the species of greatest medical importance in Brazil, *Leishmania (V.) braziliensis* (strain: MHOM/BR/1975/M2904, refereed to in the text and figures as *L.b*. 2904) and *Leishmania (L.) infantum* (strain: MHOM/BR/1972/BH46, refereed to in the text and figures as *L.i*. BH46). We also used a wild strain of *Leishmania (L.) infantum* isolated from dogs naturally infected by *L. infantum* from the endemic region of Governador Valadares, MG, Brazil (refereed to in the text and figures as *L.i*. wild). The well established laboratory strains of *L. infantum* and *L. braziliensis* were chosen to allow comparison with previous studies, as well as to choose strains that are considered reference strains for the two polar disease inducing *Leishmania*. The wild strain of *L. infantum* was chosen to compare the characteristics induced by the laboratory strain with a more recently isolated strain. Parasites were grown in Schneider medium (Sigma-Aldrich) pH 7.2 containing 10% fetal bovine serum and 1% antibiotic (penicillin 200 U/ml). All experiments were carried out between the fifth and seventh passage of parasite culture. Cultures were carried out in plastic bottles in a biochemical oxygen demand (BOD) incubator at 23°C. Cultures were monitored daily and parasites were harvested at the stationary phase for *in vitro* infection.

### Infection of PBMC with Different Strains of *Leishmania*

**Figure 1:**
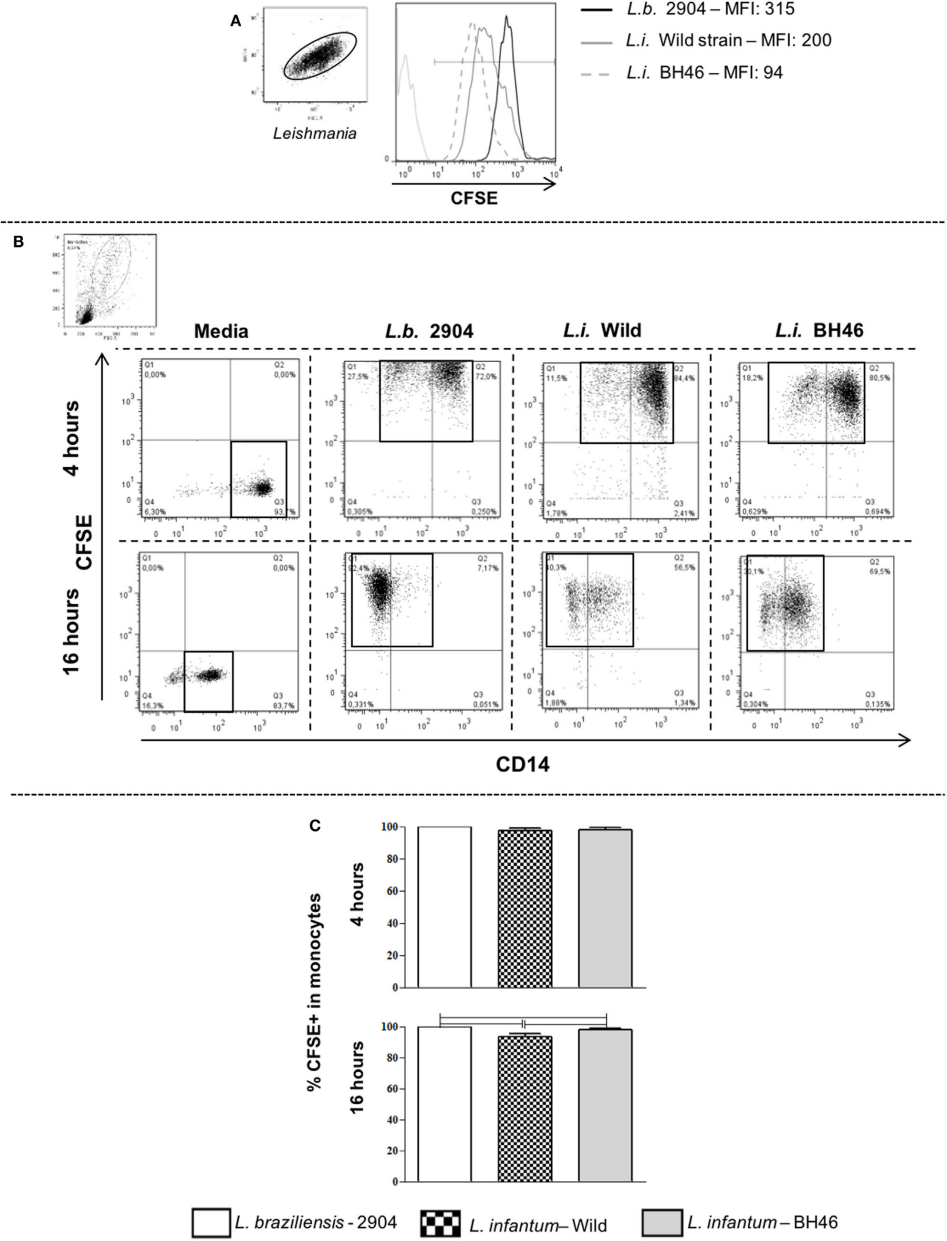
Gating strategy and determination of infection intensity of human peripheral blood mononuclear cell (PBMC)-derived monocytes using carboxyfluorescein diacetate succinimidyl ester (CFSE)-labeled parasites. Panel **(A)** shows a representative staining and mean fluorescent intensity (MFI) of *Leishmania* stained with CFSE, a green fluorescing intracellular dye. Stained *Leishmania* are displayed and gated on SSC vs. FSC parameters and then the MFI is determined in a FL1-green fluorescence histogram. The MFI for *L.b*. 2904 is intrinsically higher than that seen for *L.i. wild* or *L.i*. BH46. Panel **(B)** shows the CFSE vs. CD14 dot plots used to delineate quadrants for determining the percentage of infected monocytes. The higher intensity of CFSE staining seen in *L.b*. 2904 is reflected in the fluorescent intensity of infection with the three strains, however, by gating on total CFSE+ events, the percentage of infected cells can be determined. This is a representative experiment using one donor (*n* = 6). Following 4 or 16 h of *Leishmania* infection of PBMC with three strains, the percentage of infected monocytes was determined using flow cytometry. Panel **(C)** shows the percentage of infected monocytes at 4 h as determined by CFSE+ monocytes (*n* = 4 donors). Panel **(C)** shows the percentage of infected monocytes at 16 h as determined by CFSE+ monocytes (*n* = 6 donors). Results are expressed as mean ± SD. The horizontal brackets indicate a statistically significant difference (*p* ≤ 0.05) between the pairs, using paired one-way analysis of variance test with Tukey correction for multiple comparisons.

Promastigote forms from the different *Leishmania* isolates were labeled with the dye carboxyfluorescein diacetate succinimidyl ester (CFSE—Molecular Probes C1157), as previously described by us (31, 32). Briefly, *Leishmania* parasites were resuspended at 6 × 10^7^ parasites/ml and CFSE was added to the tube in the final concentration of 5 µM. Samples were incubated in CO_2_ incubator at 37°C for 15 min. Then, the parasites were washed three times by centrifugation at 200 *g* with ice-cold PBS plus 10% inactivated fetal calf serum (Sigma Aldrich Chemicals, St. Louis, MO, USA), and resuspended in cRPMI. Parasites alone where then run on the flow cytometer to determine staining efficiency and intensity (Figure 1).

A proportion of 10 CFSE-labeled parasites/cell was used for *in vitro* infection of individual donor cells. The frequency of metacyclic promastigotes ranged between 78 and 81% in all preparations for all the isolates evaluated here as determined by morphologic characteristics, as well as flow cytometry profiles (33). The choice of 10 parasites/cell was chosen to allow for uniform interaction between parasites and host cells (as seen in Figure 1) and thereby remove possible experimental variation due to differential intensities of parasite-cell interactions. Cultures of PBMC and parasites, or PBMC alone, were carried out using cRPMI media, in a humidified incubator at 5% CO_2_ at 37°C. After 4 h of culture, cells were run on the flow cytometer for determination of initial infection intensity by determining the percentage of infected monocytes (Figure 1). After approximately 12 h of culture, Brefeldin A was added (1 µl/ml) to samples, which were re-incubated for an additional 4 h, totaling 16 h of culture. After infection, cells were washed with PBS by centrifugation (200 *g*, 10 min at 4°C) and resuspended in PBS at a concentration of 10^7^ cells/ml for subsequent plating and staining. A total of 12 donors in five different experiments were used for these studies.

### Staining of Surface and Intracellular Molecules

The 2 × 10^5^ PBMC/well for each donor and each culture condition, obtained as described above, were incubated in a 96-round-bottom well plate with monoclonal antibodies directed against surface molecules (CD14, CD80, CD86, HLA-DR, TLR2), labeled with different fluorochromes, for 15 min at 4°C. Once labeled, the samples were washed in PBS and fixed by incubation with 200 µl of PBS containing 2% formaldehyde for 20 min. Thereafter, cells were washed with PBS, permeabilized with a 0.5% saponin-PBS solution for 15 min at 4°C, centrifuged, and incubated with monoclonal antibodies for intracellular molecules (TNF-α, IL-10, and TLR9) for 30 min at 4°C. Cells were then washed twice with saponin-PBS, resuspended in PBS and acquired using a BD FACSCanto II equipped with BD FACSDiva 6.0 software (Becton, Dickinson and Company, San Jose, CA, USA). We acquired a minimum of 30,000 gated events for each sample. All monoclonal antibodies, including isotype controls used in all reactions, were from BioLegend (San Diego, CA, USA). A total of six donors in three different experiments were used for these studies. After acquisition of the samples, the data were analyzed using the FlowJo 7.6.5 program.

### Leishmania Control Assays

The 2 × 10^5^ PBMC from four donors in two different experiments were cultured with 2 × 10^6^ *Leishmania* of each strain as performed for the cultures used to determine monocyte surface molecule and cytokine expression. At the same time point used for these measurements (16 h), cultures were centrifuged at 200 *g* for 10 min at 4°C and resuspended in 300 μl of Schneider’s media and placed in the first well of a 96-well flat bottom plate. Following this, the wells were used to begin 1:3 serial dilutions by transferring 100 μl from the first well into 200 μl of Schneider’s media in the adjacent well, and continuing down the plate until the 24th well. The plates were then incubated in the BOD incubator at 23°C and after five days of culture the plates were scored for the last dilution with detectable *Leishmania* growth using an inverted light microscope. Controls of *Leishmania* alone were used, and no significant difference was seen between the three strains in their growth upon 1:3 dilutions. This was used to calculate the fold difference in *Leishmania* between cultures infected with the different strains. The growth seen from *L. braziliensis*-infected cells was set as 1 to calculate a “*Leishmania* control index” with respect to *L. braziliensis* (which had growth up through the dilution 1:2,187).

### Statistical Analysis

One-way paired analysis of variance (ANOVA), followed by multiple comparison test of Tukey, was applied to all comparisons, as they followed a normal distribution. Pearson test was used for all correlation analysis. Data were considered significant when the *p*-value was ≤0.05. The statistical analysis was performed using the GraphPad Prism 5.0^®^ software. The principal component analysis (PCA) and cluster analysis with heatmaps were performed using the Clustvis software. The hierarchical clustering was performed using the clustering distance set as Pearson correlation subtracted from 1 and using the average distance of all possible pairs for the linkage method (34).

## Results

### *L. braziliensis* Strains Presented Greater Intensity of Staining CFSE when Compared with *L. infantum* Strains

In order to determine the infectivity profile of each *Leishmania* isolate, we labeled promastigotes from *L. braziliensis*-2904 (*L.b*. 2904), *L. infantum*-Wild (*L.i. wild*), and *L. infantum*-BH46 (*L.i*. BH46) strains with CFSE and incubated with PBMC *in vitro*. After 4 h of infection, the cells were washed and stained for anti-CD14 to determine the percent of infected cells immediately following infection (Figure 1). In addition, parallel cultures were left for a total of 16 h of infection and used for the subsequent measurements of surface molecule expression, cytokine production, and *Leishmania* control. Dot plots of CFSE vs. CD14 reveal the presence of CD14− and CD14+ phenotypes following interaction with *Leishmania*, however, the analysis of the markers were performed in the total CFSE+ monocyte population independent of CD14 expression, since it was seen that there was no major differences in the overall profiles of expression of molecules between these two subpopulations. Interestingly, the *L. braziliensis*-infected monocytes showed a more dramatic downregulation of CD14 when compared with those infected with the *L. infantum* strains (CD14-CFSE+ monocytes: *L.b*. 2904, 41.6 ± 14.9%; *L.i*. wild, 19.4 ± 7.6% and *L.i*. BH46, 21.0 ± 3.0%). Infection was measured as the percent of CFSE+ cells within the monocyte gate (Figure 1B). After 4 h of infection the percentage of CFSE+ monocytes by *L. braziliensis* (99.6 ± 0.2), and the *L. infantum* strains, *L.i*. BH46 (97.6 ± 1.7%) and *L.i*. wild (97.5 ± 1.2%), was statistically equivalent (Figure 1C). However, following 16 h slight differences in the percent of CFSE+ monocytes were seen (Figure 1C). The difference in intensity of CFSE staining on infected monocytes seen between the *L. braziliensis* strain (*L.b*. 2904) and the *L. infantum* strains was due to the greater intensity of staining of *L.b*. 2904 as seen in Figure 1A, and did not reflect a substantial difference in the percentage of infected monocytes between the three strains when considering all CFSE+ infected monocytes (Figure 1).

### Human Monocytes Infected with *L. infantum* Strains Display Lower Expression of Activation Molecules when Compared with Monocytes Infected with *L. braziliensis*

To determine the activation state of monocytes infected with the different strains, we evaluated the mean intensity fluorescence (MFI) expression of a series of activation-related molecules such as TLR2, TLR9, HLA-DR, and the costimulatory molecule ligands, CD80 and CD86, by the different cell populations after infection with *L. braziliensis* or the *L. infantum* strains.

**Figure 2:**
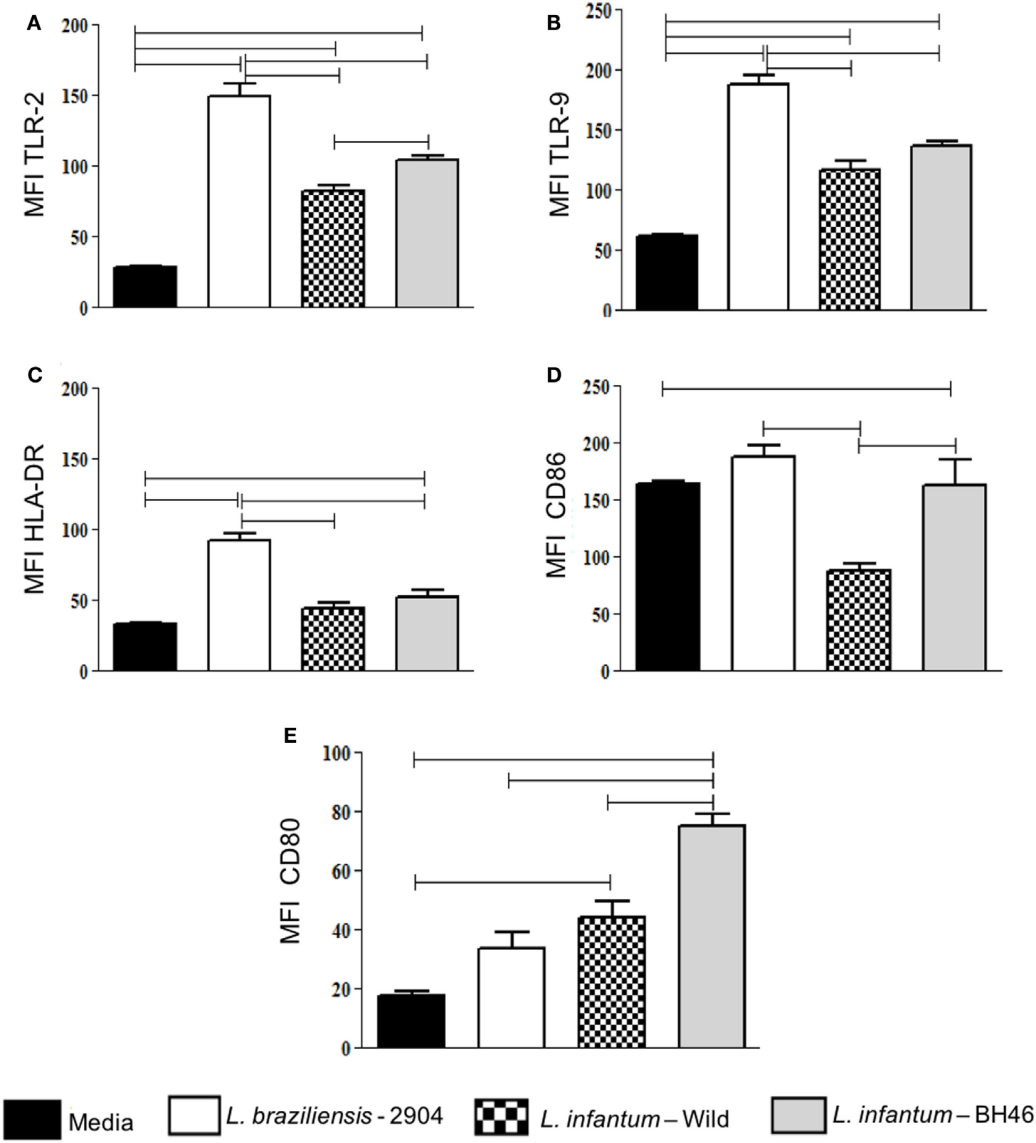
Analysis of expression of the activation-related markers TLR-2, TLR-9, HLA-DR, CD86, CD80 by monocytes before and after infection with different *Leishmania* isolates. Molecule expression as determined by calculation of mean flourescent intensity (MFI) using flow cytometry and gating on the corresponding infected monocyte population. Expression of TLR-2 **(A)**; TLR-9 **(B)**; HLA-DR **(C)**; CD86 **(D)**; and CD80 **(E)** in monocytes. Results are expressed as mean ± SD (*n* = 6 donors). The horizontal brackets indicate statistically a significant difference (*p* ≤ 0.05) between the pairs, using paired one-way analysis of variance test with Tukey correction for multiple comparisons.

With the exception of CD80 expression, infection by *L. braziliensis* leads to a higher intensity of expression of all analyzed molecules when compared with *L. infantum* strains in monocytes (Figures 2A–D). Expression of CD80 in monocytes was higher in *L. infantum* strains when compared with non-infected, while *L. braziliensis*-infected cells did not upregulate CD80 significantly (Figure 2E). Interestingly, CD86 was lower in monocytes infected with *L.i. wild* when compared with the other strains, including *L.i*. BH46 (Figure 2D).

Thus, in summary, *L. braziliensis* increases the expression of activation molecules in both infected monocytes when compared with the *L. infantum* strains, and *L.i. wild* leads to a lower expression of activation molecules, when compared with *L.b*. 2904 and to *L.i*. BH46.

### Infection of Human Monocytes with *L. infantum* Strains Leads to Less Production of TNF-Alpha and a Lower TNF-Alpha/IL-10 Ratio, when Compared with *L. braziliensis*

After observing the changes in the phenotypic expression of activation markers, which were generally reduced by infection with *L. infantum*, we sought to determine if the infection with the different isolates led to a differential expression of immunoregulatory cytokines by monocytes upon infection.

**Figure 3:**
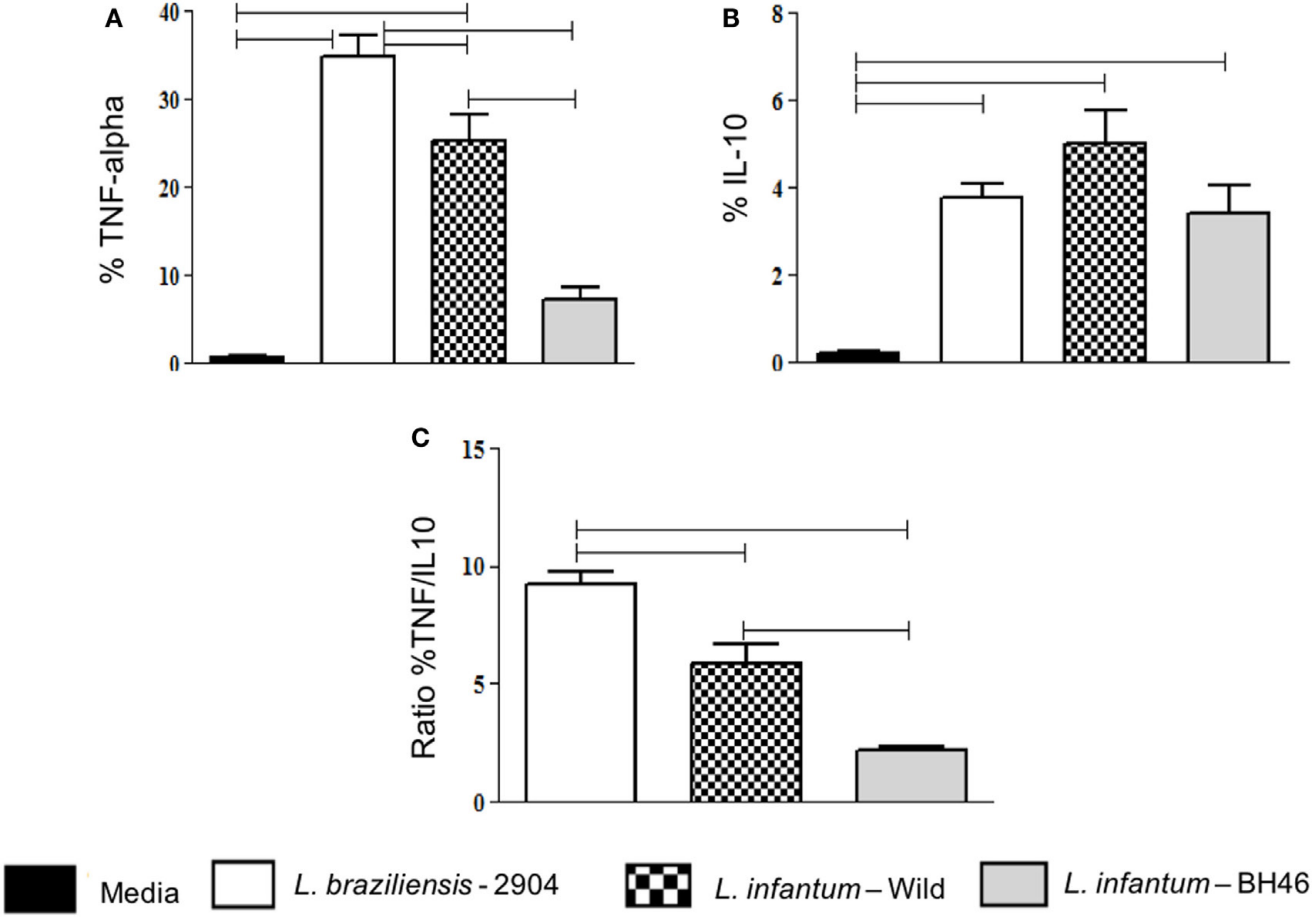
Analysis of the frequency of monocytes expressing the immunoregulatory cytokines TNF-alpha and IL-10, before and after infection with different *Leishmania* isolates. The percentage of cytokine expressing infected monocytes was determined by gating on the corresponding population. Expression of TNF-alpha **(A)**; IL-10 **(B)**; and ratio of the percent of TNF-alpha/the percent of IL-10 expression in monocytes **(C)**. Results are expressed as mean ± SD (*n* = 6 donors). The horizontal brackets indicate statistically a significant difference (*p* ≤ 0.05) between the pairs, using paired one-way analysis of variance test with Tukey correction for multiple comparisons.

Our results showed that *L. braziliensis* induced higher frequency of monocytes expressing TNF-alpha when compared with the *L. infantum* strains (Figure 3A). When we compared IL-10 expression was perceived that all strains induced an increase in IL-10 expression when compared with media, however, there was no statistical difference between the strains (Figure 3B).

To gain a better measure of immunoregulatory balance, we analyzed the TNF-alpha/IL-10 ratio and found that both *L. infantum* strains induced a lower TNF-alpha/IL-10 ratio in monocytes when compared with infection with *L. braziliensis* (Figure 3C). Comparing the two isolates of *L. infantum*, we observed that the TNF-alpha/IL-10 ratio was lower in infected monocytes with *L.i*. BH46 when compared with *L.i. wild* (Figure 3C).

In addition, we measured IL-12/p40 expression and found that there was no difference between monocytes infected with the different *Leishmania* isolates. All induced high levels of IL-12/p40 expression (data not shown).

**Figure 4:**
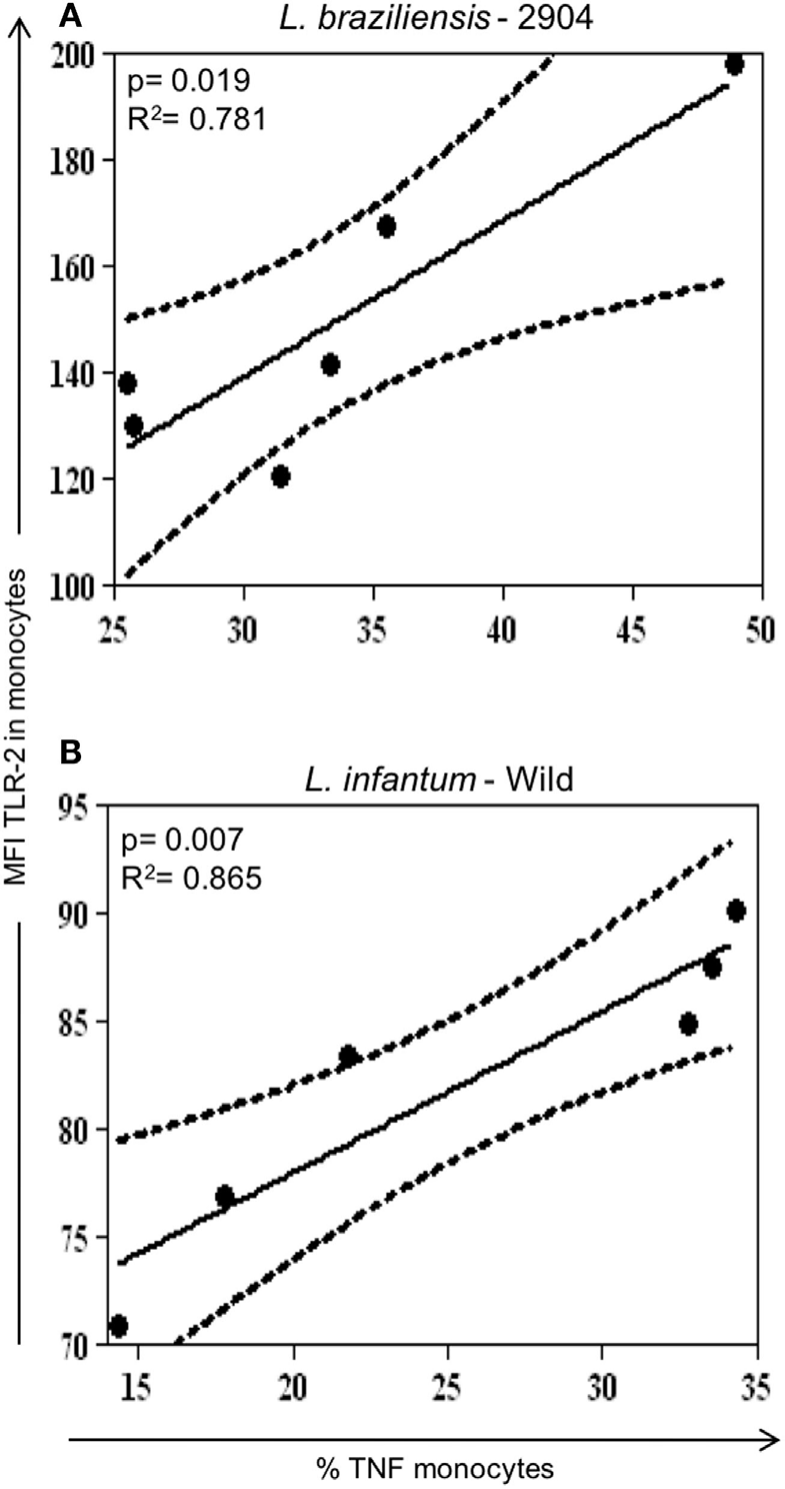
Correlation analysis between expression of TLR2 with TNF-alpha in monocytes infected with different *Leishmania* isolates. Results are expressed as scatterplots, with each point representing an individual donor, showing statistically significant difference (*p* ≤ 0.05) and *R*^2^ value between different strains. Correlation analyses were performed using Pearson’s correlation coefficient (*n* = 6 donors). **(A)** Positive correlation between TLR2^+^/TNF-alpha^+^ in monocytes infected with *L. braziliensis*. **(B)** Positive correlation between TLR2^+^/TNF-alpha^+^ in monocytes infected with the different *Leishmania* strains.

Seeking to determine whether there was relationship between the expression of activation molecules expression and cytokine production, correlation analyzes were performed between these variables. A positive correlation between the expression of TLR2^+^/TNF-alpha^+^ was observed in monocytes when infected with the *L. braziliensis* strain (Figure 4A) and *L.i. wild* (Figure 4B). No other positive or negative correlations were found between other activation molecules and cytokines (not shown).

### Principle Component Analysis and Cluster/Heatmap Highlight Distinct Monocyte Responses upon Infection with *L.b*. 2904 vs. *L.i*. wild and *L.i*. BH46

**Figure 5:**
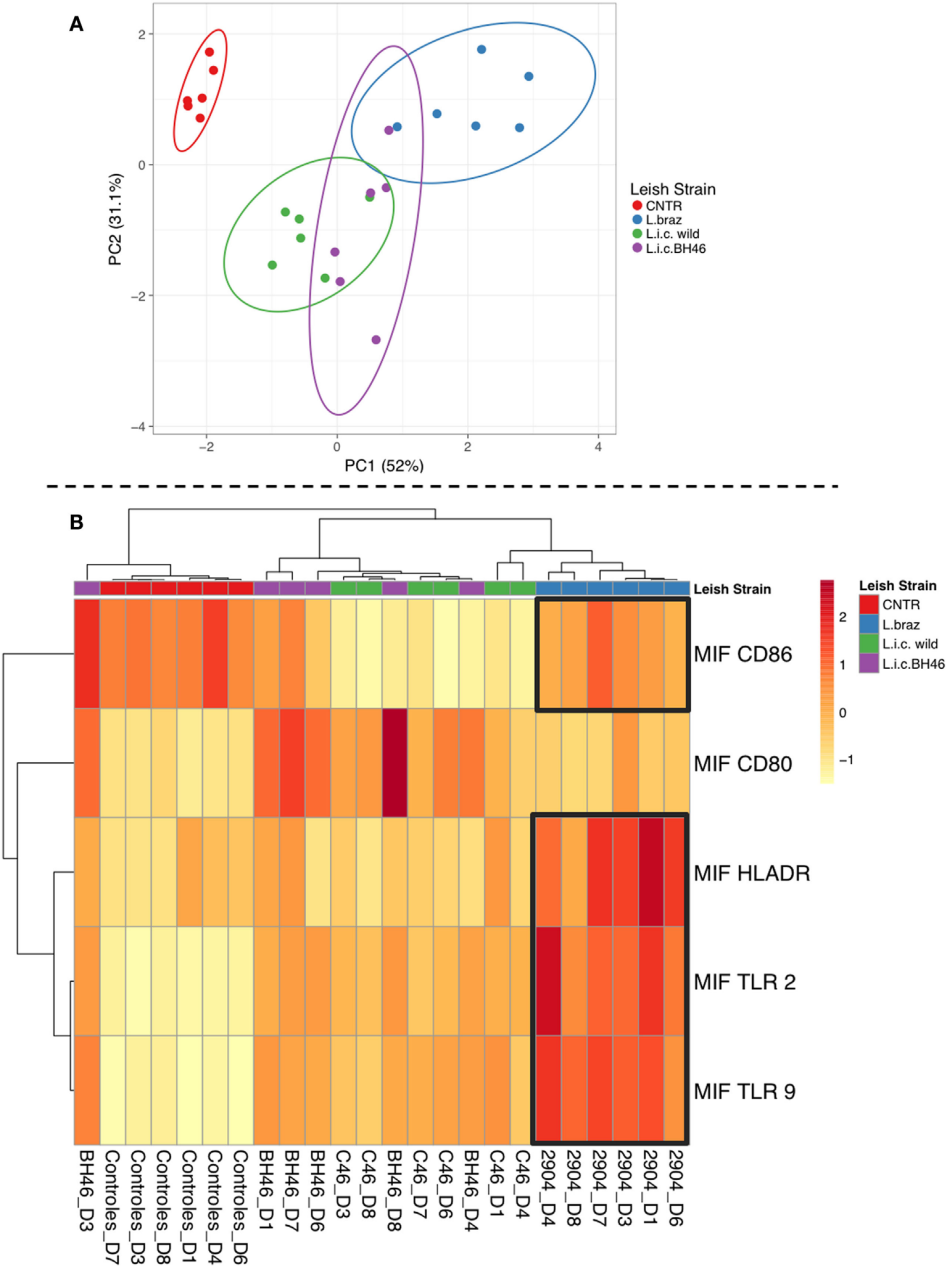
Principal component analysis and cluster/heatmap analysis using overall immune profiles of monocytes infected with different *Leishmania* strains. Panel **(A)** shows the principle component analysis shows that PC1 (responsible for 52% of the variation) and PC2 (responsible for 31.1% of the variation) segregate the populations in a 2D plot. The non-infected monocytes form a separate populations, while the *Leishmania*-infected populations form partially overlapping groups with *L.i*. wild and *L.i*. BH46 showing the greatest overlap, and *L. braziliensis*-infected monocytes partially overlapping with *L.i*. BH46. Panel **(B)** shows the cluster analysis and heatmap analysis which clearly segregates into a tree containing the *L. braziliensis* population, defined by high expression of HLA-DR, TLR2, TLR9, and CD86, and lower expression of CD80, while the *L. infantum*-infected populations share a tree separate from the non-infected cluster and the *L. braziliensis*-infected cluster. There are three outliers, two from the *L.i*. wild group that form a branch from the *L. braziliensis* tree, and one *L.i*. BH46 individual that forms a branch from the non-infected tree. Analysis was perfumed as indicated in Section “Materials and Methods” using the Clustvis software.

Having demonstrated clear differences between the monocyte response upon infection with *L.b*. 2904 vs. *L.i*. wild and *L.i*. BH46, we performed PCA associated with cluster/heatmap representations to reveal monocyte profiles defined by infection with different *Leishmania* strains, and determine to what extent they segregate into distinct or overlapping populations based on these global immune profiles. As seen in Figure 5A, the PCA resulted in PC1 (52% of the variation) and PC2 (31.1% of the variation) separating the different populations on the 2D plot. The non-infected population is non-overlapping and separate from all three of the *Leishmania*-infected populations. The *Leishmania*-infected monocyte populations formed three overlapping groups, with the two *L. infantum* strains displaying the greatest overlap, and with the *L. braziliensis*-infected monocyte population overlapping only with the *L.i*. BH46 population. Further analysis of the data to reveal associations between the groups using the cluster analysis and heatmap show that the *L. braziliensis*-infected monocytes cluster together with a clear association of higher levels of HLA-DR, TLR2, TLR9, and CD86 impacting the formation of this cluster (Figure 5B). Interestingly, the patterns of expression of the same molecules, along with a higher expression of CD80, formed a major cluster tree including members from both the *L.i*. BH46 and *L.i*. wild-infected monocytes. This indicates that these populations are more similar to one another based on their global immune profile. These findings reflect the greater overlap between the two *L. infantum*-infected monocytes seen in the PCA analysis. Finally, there were two individuals from the *L.i*. wild group that formed a sub-branch under the *L. braziliensis* tree, and one *L.i*. BH46 individual that segregated in a sub-branch of the non-infected monocytes (Figure 5B).

### *L. infantum*-Infected Monocyte Cultures Were Greater than a 100-Fold Less Effective at Controlling *Leishmania* than Those Infected with *L. braziliensis*

To determine the functional consequences of the down modulatory profile of monocytes infected with *L. infantum* (less activation and less inflammatory cytokine production) when compared with *L. braziliensis*-infected monocytes, we measured the ability of cultures to control *Leishmania* parasites following the overnight cultures.

Infected monocyte cultures from four donors were submitted to serial dilutions in Schneider’s media to determine relative ability to control *Leishmania* in cultures infected with the three strains. The last *Leishmania* positive well was scored as 1 from cultures infected with *L.b*. 2904 (dilution 1:2,187) and the fold increase in *Leishmania* calculated using the last serial dilution well with positive growth for the other two strains, *L.i*. wild and *L.i*. BH46. Using four different PBMC donors the average relative *Leishmania* growth from cultures infected with *L.i*. wild (dilutions 1:531,441–1,594,323) and *L.i*. BH46 (dilutions 1:177,147–531,441) were on average 607-fold and 122-fold higher, respectively, when compared with *L.b*. 2904. Thus, while given equivalent initial infections, the *L. infantum*-infected monocyte cultures were over 100-fold less effective at controlling *Leishmania* than *L. braziliensis*-infected cultures (Table 1).

**Table 1:** Monocytes infected by *L. infantum* strains are greater than 100-fold less effective at controlling *Leishmania*.

	*L.b*. 2904	*L.i*. BH46	*L.i*. wild
Fold dilution difference^a^	1	607 ± 143	122 ± 48

*^a^PBMC from four donors were infected as described in Section “Materials and Methods” and then submitted to serial dilution in Schneider’s media. The last dilution with positive Leishmania growth was set at 1 and used to calculate the fold increase in dilution with positive Leishmania for the other strains. The data are represented as average ± SE from four separate cultures*.

## Discussion

This work was designed to investigate whether distinct species and strains of *Leishmania* induce differential activation upon infection of human monocytes *in vitro*. Our hypothesis was that if there were differences in the response induced by the different strains, that they could help explain distinct immune responses and subsequent clinical forms observed in human infection with *L. braziliensis* (potential for tegumentary forms of disease) vs. *L. infantum* (potential to develop visceral disease). First, we investigated the infection intensity and its relation to monocyte infected by different strains. For this, the infectivity of the strains was characterized using CFSE-labeled promastigotes and analyzing the percentage of infected monocytes as those expressing fluorescence due to CFSE-labeled *Leishmania* (medium to high intensity). Previous studies have shown that *Leishmania* promastigote-macrophage interaction can be measured when the parasites are stained with CFSE and analyzed by flow cytometry (31, 32). While there is a range of intensities of CFSE+ *Leishmania*-infected monocytes between the strains, the percentage of infected monocytes was equivalent between the strains at 4 h following infection (Figure 1) and a very slight difference at 16 h. Interestingly, the apparent increase in infection intensity (MFI) by *L. braziliensis* was due to an inherent increased intensity of CFSE staining of that strain when compared with the *L. infantum* strains (Figure 1). Thus, the relative infection intensity and percentage of infected monocytes was equivalent between the strains, and differences in infection intensity do not explain the differences seen in activation, cytokine profiles and *Leishmania* control. While there were no significant differences between infection intensity of the strains, it is well known that the surface of different species of *Leishmania* is decorated with numerous GPI-linked complex molecules that are associated with interaction, survival and virulence in the host cell (5, 27). Glycoprotein 63 and the lipophosphoglycan (LPG) are two of the most abundant molecules, which are primarily responsible for the virulence of the parasite (27, 35). The existence of differences between the intra- and inter-*Leishmania* species structure of these molecules may influence the parasite–host cell interaction and, in turn, the establishment of infection (28, 29).

Several studies have demonstrated that human monocytes/macrophages display phenotypic and functional heterogeneity upon activation (36), and thus, in our study we observed the formation of two subpopulations of monocytes based on expression of the CD14 marker. The expression of the different molecules in these subpopulations was evaluated, however, no difference was observed in the expression of the molecules.

*Leishmania braziliensis* indeed leads to higher expression of activation molecules such as TLRs, HLA-DR, and costimulatory ligands, when compared with the infected monocytes with *L. infantum* isolates. The fact that the *L. infantum* isolates lead to an even lower expression of the activation markers, especially TLR2, TLR9 and HLA-DR, when compared with *L. braziliensis*, are also in accordance with the fact that this parasite triggers a downregulated cellular response *in vivo* in experimental models, as well as in human disease (37, 38). Expression of the costimulatory molecule ligands, CD80 and CD86, which are primarily expressed on the surface of antigen presenting cells further support this hypothesis. These molecules are responsible for delivering the second signal for the activation and proliferation/control of T lymphocytes through binding to their receptors, CTLA-4 and CD28, respectively (39). Interestingly, we found that monocytes infected with the *L.i. wild* strain displayed a lower expression of CD86. Strikingly, when we evaluated the expression of CD80, we observed that the *L.i*. BH46 strain induced a higher expression of this molecule on monocytes compared with the other isolates, as well as with the control. It has been shown that CD80 binds preferentially to CTLA-4 leading to an inhibition of lymphocytes, while CD86 binds preferentially CD28, promoting lymphocyte activation (40, 41). Therefore, the lower expression of CD86 and the higher expression of CD80 by monocytes infected with *L.i. wild* and L. c. BH46, may contribute to a low T cell response observed in *L. infantum* infection. This conclusion is also supported by others who found that canine monocytes infected with *L. infantum* display a decreased expression of costimulatory molecules, thus undermining the activation (42). A decreased T lymphocyte activation, possibly caused by a decrease in CD86 and an increase in CD80 expression, is consistent with the findings observed in human visceral leishmaniasis (41).

TLRs are responsible for specific recognition of pathogen-derived antigens, and can alter the expression of immunoregulatory cytokines (43). TLR2, TLR4 and TLR9 are the main receptors involved in the recognition of *Leishmania* molecules (12, 26, 43, 44). In this study, we observed that monocytes infected with *L. braziliensis* showed increased expression of TLR2 and TLR9 compared with those infected with the *L. infantum* strains. Becker et al showed that *L. major* LPG can be recognized by TLR2 on NK cells, trigger cell activation and further increase the levels of TLR2 on its surface (45). LPG purified from *L. major* has been proven to be the main TLR2 agonist, since this receptor participates in the recognition of *Leishmania* and activation of an important pathway in infection control (46). Thus, it is possible that TLR2 is also involved in *L. braziliensis* recognition. Few studies have evaluated TLR expression in visceral leishmaniasis, therefore, our results showing the increase of TLR2 after infection of monocytes by *L. infantum*, when compared with non-infected cultures, indicate that these receptors may also be involved in the recognition of the parasite (47, 48). TLR9 is essential for disease control and parasite replication in experimental models. TLR9 deficient mice develop severe lesions and have high parasite load when infected with *L. braziliensis* (49). Studies in human disease have shown that patients with visceral leishmaniasis present an increase in TLR4 and TLR9 expression after treatment with miltefosine, generating a strong proinflammatory response which is important in disease resolution (50). Moreover, monocytes from patients with cutaneous leishmaniasis due to *L. braziliensis* infection display a high expression of TLR9, suggesting a role for this molecule in active disease (12). Here, we observed that infection with *L. braziliensis* leads to a higher expression of TLR9, when compared with *L. infantum* strains. This fact may favor the generation of a more effective inflammatory response at *Leishmania* killing.

A protective immune response against leishmaniasis has been associated with an efficient cell response resulting in the production of cytokines such as IL-12, IFN-gamma, and TNF-alpha that leads to activation of macrophages and parasite elimination (51). However, there is an association of increased expression of IFN-gamma, activated T cells and monocytes, and lesion size in cutaneous leishmaniasis, indicating that despite the fact that a strong inflammatory response is important for parasite control, it can also lead to tissue destruction (10, 12, 14). In contrast, a modulatory immune response, characterized by the production of IL-10, is a hallmark of visceral leishmaniasis (23, 52). In this study, we evaluated the expression of TNF-alpha and IL-10 by infected monocytes, to determine if the different strains would lead to distinct functional cytokine expression, which could influence the overall environment and, thus, the subsequent immune response. Monocytes infected by *L. braziliensis* showed an increased expression of TNF-alpha compared to monocytes infected by the *L. infantum* strains. Between monocytes infected with *L.i.c* strains, the wild strain showed a higher TNF-alpha expression in monocytes infected. Interestingly, the L.i. wild strain also showed a positive correlation between the expression of TNF-alpha and TLR2, as did the *L. braziliensis* strain. This is one of the few characteristics that was similar between the *L.i*. wild and *L. braziliensis* strain (the other was CD80 expression). The higher expression of TNF-alpha by the *L.i. wild* strain is unexpected and further molecular characterization of this isolate may clarify this question. A clear increase in IL-10 expression was observed after infection with all isolates in monocytes, even more pronounced by the infection with *L.i. wild* strain. Despite the importance of the expression of the individual cytokines, the balance between the production of inflammatory versus anti-inflammatory cytokines provides important information with respect to the predominant microenvironment. When we analyzed the TNF-alpha/IL-10 ratio, we observed that *L. braziliensis*-infected monocytes displayed an approximately twofold higher ratio, indicating a greater inflammatory environment, when compared with that induced by *L. infantum*-infected monocytes. Finally, the PCA analysis (Figure 5A) shows the global immune profile of the monocyte populations infected with different *Leishmania* strains form populations distinct from the non-infected monocytes with PC1 and PC2 accounting for 83% of the variation. Interestingly, the two L. infantum populations display the greatest overlap and the *L. braziliensis* population only overlaps slightly with the *L.i*. BH46 population. When performing a cluster and heatmap analysis, again the global immune profile segregates the *L. braziliensis*-infected monocytes from the *L. infantum*-infected groups, with the exception of three outliers (Figure 5B).

Taken together these findings are consistent with the characteristics observed in human infection, in which patients with tegumentary forms of leishmaniasis due to *L. braziliensis* infection display an inflammatory response with response-associated pathology, while patients with visceral disease display a downmodulated environment. The use of this relatively reductionist model of *in vitro* infection, suggests that the type of immune response observed in tegumentary and visceral leishmaniasis (higher activation and inflammatory profile versus lower activation and less inflammatory, respectively) can be defined early on. The identification of the parasite molecules responsible for these differences in the activation process is an important point to be addressed. Furthermore, strategies that can interfere with this response at early stages may be beneficial for disease control.

## Ethics Statement

This study was carried out in accordance with the recommendations of CONEP with written informed consent from all subjects. All subjects gave written informed consent in accordance with the Declaration of Helsinki. The protocol was approved by the National Ethical Committee (CONEP # CAAE 01229212.0.0000.0049).

## Author Contributions

AV and KG contributed to design of the work, acquisition, and analysis/interpretation of data, drafting the work, revising it critically, and final approval of the version to be published and is in agreement to be accountable for all aspects of the work; LM and RG contributed to design of the work, acquisition, revising it critically, and final approval of the version to be published; WD contributed to design of the work, analysis/interpretation of data, drafting the work, revising it critically, and final approval of the version to be published.

## Conflict of Interest Statement

The authors declare that the research was conducted in the absence of any commercial or financial relationships that could be construed as a potential conflict of interest.
